# Baseline Oral Behaviours in Relation to Follow-Up Temporomandibular Joint Disease Severity After Intra-Articular I-PRF: A Baseline-Adjusted Exploratory Cohort Study

**DOI:** 10.3390/jcm15103575

**Published:** 2026-05-07

**Authors:** Marcin Sielski, Maciej Chęciński, Kamila Chęcińska, Natalia Turosz, Dariusz Chlubek, Maciej Sikora

**Affiliations:** 1 Department of Maxillofacial Surgery, Hospital of the Ministry of the Interior and Administration, Wojska Polskiego 51, 25-375 Kielce, Poland; marcinsielski@gazeta.pl (M.S.); maciej.checinski@pimmswia.gov.pl (M.C.); natalia.turosz@pimmswia.gov.pl (N.T.); maciej.sikora@pimmswia.gov.pl (M.S.); 2 National Medical Institute of the Ministry of the Interior and Administration, Wołoska 137 Str., 02-507 Warsaw, Poland; 3 Department of Biochemistry and Medical Chemistry, Pomeranian Medical University, Powstańców Wielkopolskich 72, 70-111 Szczecin, Poland; dchlubek@pum.edu.pl

**Keywords:** temporomandibular joint disorders, temporomandibular joint, injections, intra-articular, fibrin, platelet-rich

## Abstract

**Background/Objectives:** Intra-articular injectable platelet-rich fibrin (I-PRF) is increasingly used in the management of joint-origin temporomandibular joint (TMJ) disorders, but variability in follow-up TMJ disease severity remains substantial. This study assessed whether baseline oral behaviors were associated with follow-up TMJ disease severity after intra-articular I-PRF therapy, using baseline-adjusted analyses of follow-up HI. **Methods:** This secondary exploratory cohort analysis was conducted within the framework of a registered clinical trial (NCT05883982). Fifty-one consecutive adult patients treated with intra-articular I-PRF were included. Baseline oral behaviors were assessed using the Oral Behaviors Checklist (OBC-21), and TMJ disease severity was quantified using the Helkimo Index (HI) at baseline and final follow-up. Associations between baseline predictors and follow-up HI were evaluated using baseline-adjusted linear regression models including baseline HI as a covariate. **Results:** Mean (SD) HI decreased descriptively from 7.0 (6.2) at baseline to 2.5 (3.0) at final follow-up (*p* < 0.001). Baseline HI was strongly associated with follow-up HI (β = 0.32, 95% CI 0.22 to 0.41, *p* < 0.001), whereas symptom duration (β = 0.02, 95% CI −0.10 to 0.15, *p* = 0.70) and injection laterality (β = −0.42, 95% CI −1.73 to 0.89, *p* = 0.52) were not. No OBC-21 item showed a clear baseline-adjusted association with follow-up HI after correction for multiple comparisons. The largest absolute baseline-adjusted regression coefficients were observed for awake bruxism (β = 0.80, 95% CI 0.08 to 1.52, FDR-adjusted *p* = 0.60) and singing (β = −0.58, 95% CI −1.41 to 0.25, FDR-adjusted *p* = 0.83). **Conclusions:** Baseline oral behaviors did not emerge as strong standalone baseline-adjusted factors associated with follow-up HI after intra-articular I-PRF therapy. However, the observed associations may still have been influenced by baseline disease severity dimensions not fully captured by total baseline HI, such as structural disease stage, pain intensity, psychosocial burden, or other unmeasured clinical severity features. The observed nominal and directional item-level patterns should be interpreted only as hypothesis-generating and require confirmation in larger, better-characterized cohorts.

## 1. Introduction

Temporomandibular disorders (TMDs) are among the most common causes of chronic orofacial pain [1,2]. In addition to pain, they result in functional limitations, manifesting as limited jaw mobility, difficulty chewing food, difficulty speaking, and difficulty with daily activities [3,4]. These difficulties translate into a reduction in health-related quality of life [3,4].

The group of diseases collectively referred to as TMD is broad and encompasses disorders of both the myofascial system and the temporomandibular joint (TMJ) [5,6]. Temporomandibular joint disorders (TMJDs) are divided into the following main categories: (a) internal derangement, (b) osteoarthritis, (c) degenerative joint disease, and (d) habitual dislocation. The first three may be successive stages of the same disease [5,6].

TMDs have a diverse etiology and clinical course and therefore require a variety of therapeutic approaches, which may serve different therapeutic goals [7,8]. Minimally invasive intra-articular surgical interventions are used in TMJDs resistant to conservative treatment [9,10,11]. These include joint lavage (arthrocentesis) and injections into the joint cavity or joint soft tissue [10,11,12]. TMJ injections are a collection of heterogeneous surgical techniques that differ in protocol, which includes: (a) the site of injection; (b) the volume of the substance; (c) the number of injections; (d) the interval between injections; and (e) the substance used [13,14,15].

There is no consensus among researchers regarding the protocols used [13,14,15]. The most current scientific debate concerns the choice of injectable substances [16,17,18]. Most research concerns autologous blood products (growth factors), hyaluronic acid supplementation (the main component of synovial fluid), and anti-inflammatory drugs (both steroidal and nonsteroidal) [16,17,18,19,20,21,22]. Recently, studies have also reported on combination approaches, such as the intra-articular administration of a Platelet-Rich Plasma (PRP) and HA mixture [22,23,24]. Injectable platelet-rich fibrin (I-PRF) is an autologous blood-derived product obtained from centrifuged venous blood and contains platelets within a fibrin-rich liquid fraction that has been proposed to support tissue repair and regenerative processes [25,26,27].

To monitor TMJ disease severity in a standardized manner, the Helkimo Index (HI) is commonly used as a composite clinical measure capturing key domains of dysfunction and symptoms [28,29,30,31]. In typical clinical implementations, component scores are combined into a total score (expressed on a 0–25 scale), where higher values reflect greater TMJ dysfunction severity. This makes HI suitable for quantifying baseline severity and follow-up TMJ disease status in a clinically interpretable way [32,33,34].

Oral behaviors assessed with the OBC-21 are considered to be associated with TMJ symptoms and functional limitations in patients with TMD, although these behaviors are increasingly interpreted within a broader biopsychosocial framework rather than solely as mechanical loading factors [35,36,37,38]. They may therefore be considered candidate baseline variables potentially associated with follow-up TMJ disease severity in patients with TMJD, although their role as predictors of treatment response remains uncertain. The Oral Behaviors Checklist (OBC-21), included among DC/TMD self-report instruments, captures the frequency of 21 behaviors spanning sleep-related behaviors and waking oral habits. In the context of secondary exploratory analyses, these behaviors may be examined as candidate baseline variables associated with follow-up TMJ disease severity rather than as established predictors of treatment response. The checklist covers (in order): sleep bruxism (B1); sleep posture with jaw pressure (B2); awake bruxism (B3); awake clenching (B4); nonfunctional tooth contact (B5); masticatory muscle tension without tooth contact (B6); jaw thrusting/shifting (B7); tongue pressing against teeth (B8); tongue held between teeth (B9); biting/chewing soft tissues (B10); jaw bracing/holding the jaw rigid (B11); biting/holding objects between teeth (B12); chewing gum (B13); playing instruments involving the mouth/jaw (B14); resting the chin on the hand (B15); unilateral chewing (B16); snacking on foods requiring chewing (B17); prolonged speaking (B18); singing (B19); yawning (B20); and cradling a phone between head and shoulder (B21) [39,40,41,42].

This secondary exploratory analysis aimed to assess whether baseline OBC-21 items were associated with follow-up TMJ disease severity measured using the HI after intra-articular I-PRF therapy, with baseline HI included as an adjustment covariate. Given the exploratory character of the study and the number of candidate variables examined, the analysis was intended to generate hypotheses regarding associations between baseline oral behaviors and follow-up TMJ disease severity rather than to support confirmatory or predictive inferences.

## 2. Materials and Methods

### 2.1. Study Design and Reporting Framework

This secondary exploratory cohort analysis was conducted within the framework of a clinical trial registered at ClinicalTrials.gov (NCT05883982), in a cohort of patients treated with intra-TMJ I-PRF. The analysis examined associations between baseline oral behaviors and follow-up TMJ disease severity, with baseline HI included as an adjustment covariate. The full clinical outcomes of the parent trial are outside the scope of this report. The manuscript was prepared in accordance with the STROBE statement, to the extent applicable.

### 2.2. Ethics and Trial Registration

Ethical approval was obtained from the Bioethics Committee at the Świętokrzyska Medical Chamber in Kielce (1/2022-VIII) prior to participant enrollment, and all participants provided written informed consent.

### 2.3. Participants

Consecutive adult patients presenting to the Department of Oral and Maxillofacial Surgery at the Ministry of the Interior and Administration Hospital in Kielce (St. John Paul II) with joint-origin temporomandibular joint (TMJ) dysfunction and accepted clinical indications for intra-articular injection therapy were screened. Patients were eligible for this secondary analysis if they received intra-TMJ I-PRF treatment and completed protocol-defined baseline and final follow-up assessments for the HI.

Inclusion criteria were: (1) age ≥ 18 years; (2) written informed consent; (3) intra-TMJ I-PRF treatment per protocol; and (4) availability of baseline OBC-21 measurements and HI values at both baseline and final follow-up. Exclusion criteria were: (1) bleeding diathesis; (2) severe psychiatric illness interfering with consent or questionnaire completion; (3) TMJ prosthesis; (4) TMJ ankylosis; and (5) skin disease involving the preauricular area on the affected side.

### 2.4. Treatment Protocol (Intra-TMJ I-PRF)

Patients were treated according to a standardized institutional protocol using intra-articular I-PRF injections into the TMJ. The injected volume per joint was 0.4 mL in five sessions performed at 14-day intervals.

At each session, autologous whole blood was collected from the antecubital vein into sterile 9 mL polycarbonate vacuum tubes intended for I-PRF/S-PRF/A-PRF preparation. The tubes were sterile, pyrogen-free, radiosterilized, and contained no chemical additives or anticoagulant. Blood samples were centrifuged immediately after collection at 630 rpm, corresponding to approximately 35× *g*, for 3 min using an iFuge D06 centrifuge (Neuation Technologies, Kalol, India) equipped with a fixed-angle rotor. Centrifugation was initiated within approximately 1 min after blood collection, and the liquid I-PRF fraction was injected within approximately 1–2 min after centrifugation.

The recipient area was disinfected and the injection point was determined using anatomical landmarks located approximately 10 mm anterior and 2 mm inferior to the tragus along the line connecting the tragus with the lateral canthus. The needle was inserted to a depth of approximately 25 mm to reach the central part of the upper TMJ compartment.

### 2.5. Timepoints and Data Collection

Data were collected at six protocol-defined timepoints during the parent trial. For the present analysis, two timepoints were used:

- tb (baseline): immediately before the first I-PRF injection;
- tf (final follow-up): the last available protocol-defined post-treatment assessment (ideally scheduled 2 weeks after the fifth injection; approximately 2.5 months after treatment initiation).

Baseline oral behaviors were assessed at tb using the OBC-21, and the HI was assessed at tb and tf. Intermediate timepoints were not analyzed in this report. Follow-up HI values were analyzed with adjustment for baseline HI values in order to account for baseline disease severity.

### 2.6. Variables and Definitions

#### 2.6.1. Outcome

- HI: Total score ranged from 0 to 25 and was computed as the sum of five components, each scored 0, 1, or 5.
- Follow-up HI (HI_tf): The primary outcome for this secondary exploratory analysis was the total HI score measured at the final follow-up timepoint (t_f).
- Baseline HI (HI_tb) was included as an adjustment variable in statistical models to account for baseline disease severity.

#### 2.6.2. Candidate Predictors and Adjustment Variables

- OBC-21: Each item captured the frequency of the specific behavior during the preceding week on an ordinal scale from 0 (“never”) to 4 (“all the time”), with an additional response option “unable to determine”. Responses marked as “unable to determine” were treated as missing for that specific item, resulting in item-specific analytic sample sizes.
- Symptom duration (years): Self-reported duration prior to treatment; summarized as mean (SD) and analyzed as a continuous variable.
- Injection laterality: Categorized as unilateral vs. bilateral for this analysis.
- Baseline HI (HI_tb): Included as an adjustment covariate in regression models to account for baseline disease severity.

### 2.7. Bias and Missing Data

Consecutive recruitment from routine clinical practice and the application of predefined eligibility criteria were intended to reduce selection bias. However, given the secondary and exploratory observational design, residual bias and unmeasured confounding remain possible. All included participants had complete HI data at baseline and final follow-up, so no imputation of outcome data was necessary.

For the OBC-21, responses marked as “unable to determine” were treated as missing for the corresponding item. Associations between individual baseline OBC-21 items and follow-up HI were estimated using available-case baseline-adjusted regression analysis, resulting in item-specific sample sizes. Missing predictor data were not imputed. The pattern of missing OBC-21 responses was examined descriptively and did not suggest a clear systematic pattern across items or participants. Baseline HI values were complete for all included participants and were included as adjustment covariates in regression analyses. Given the exploratory item-level structure of the analysis and the number of comparisons performed, the findings should be interpreted cautiously.

### 2.8. Sample Size

The sample size was determined by the number of consecutive eligible patients with HI data available at baseline and final follow-up. No a priori power calculation was performed because the analysis was secondary and exploratory.

### 2.9. Statistical Analysis

All analyses were two-sided with a nominal significance level of 0.05. Continuous variables were summarized as mean (SD). Change from baseline to final follow-up in the HI was evaluated using the Wilcoxon signed-rank test. The distributions of HI scores at baseline and final follow-up were visualized using overlaid histograms. This comparison was descriptive and not used as the primary outcome framework for association analyses.

Associations between baseline oral behaviors and follow-up HI were evaluated using linear regression models with follow-up HI (HI_tf) as the dependent variable and individual baseline OBC-21 item scores entered as candidate predictors. OBC-21 item scores were analyzed as ordinal numeric predictors reflecting increasing frequency of the reported behavior. Baseline HI (HI_tb) was included as an adjustment covariate in all models to account for baseline disease severity. Separate baseline-adjusted models were fitted for each OBC-21 item and specified as:

HI_tf ~ OBC_item + HI_tb

Symptom duration and injection laterality were evaluated using analogous baseline-adjusted regression models. Unstandardized regression coefficients with 95% confidence intervals and corresponding *p*-values were reported. Model fit and residual distributions were evaluated descriptively using residual-versus-fitted plots and inspection of residual distributions. These diagnostics were used to identify major deviations from linear model assumptions, including strong non-linearity, marked heteroscedasticity, or influential residual patterns.

The primary inferential analyses were based on baseline-adjusted regression models with follow-up HI as the outcome. Change from baseline was summarized only descriptively and was not used as the framework for predictor–outcome association analyses. This approach was chosen to reduce distortion related to mathematical coupling and regression-to-the-mean effects.

To account for multiple exploratory comparisons across OBC-21 items, false discovery rate (FDR) control using the Benjamini–Hochberg procedure was additionally applied.

### 2.10. Software

Analyses were conducted in Python (version 3.11.2; Python Software Foundation, Wilmington, DE, USA) using NumPy and pandas for data handling, SciPy for Wilcoxon signed-rank test, and statsmodels for regression analyses. Google Workspace (version 2026.01.25; Google LLC, Mountain View, CA, USA) was used for table and figure preparation.

## 3. Results

### 3.1. Participant Flow and Analytic Sample

A total of 51 consecutive patients who initiated intra-TMJ I-PRF treatment met the eligibility criteria for inclusion in this secondary analysis. Complete baseline (tb) and final follow-up (tf) HI data were available for all 51 participants, and all participants were included in the outcome analyses. Accordingly, there was no loss to follow-up with respect to the primary outcome analyzed in this report. For item-level analyses of baseline OBC-21 responses, available-case analysis was applied, resulting in item-specific sample sizes ranging from n = 42 to n = 51.

### 3.2. Baseline Characteristics and Outcome Data

The study cohort consisted of 41 women (80.4%) and 10 men (19.6%). The mean age of participants was 44.8 years (SD 14.6; range 18–81 years). Symptom duration prior to treatment averaged 5.3 years (SD 5.5; available for 48/51 patients). Baseline TMJ disease stage was described using the Wilkes classification (stages 0–5). For patient-level description, the higher stage of the two joints was used for classification. The distribution of stages was as follows: stage 2 in 31 patients (60.8%), stage 3 in 6 patients (11.8%), stage 4 in 9 patients (17.6%), and stage 5 in 5 patients (9.8%). No patients were classified as stage 0 or 1. Injection laterality was bilateral in 19/51 (37.3%), right-only in 15/51 (29.4%), and left-only in 17/51 (33.3%).

**Figure 1:**
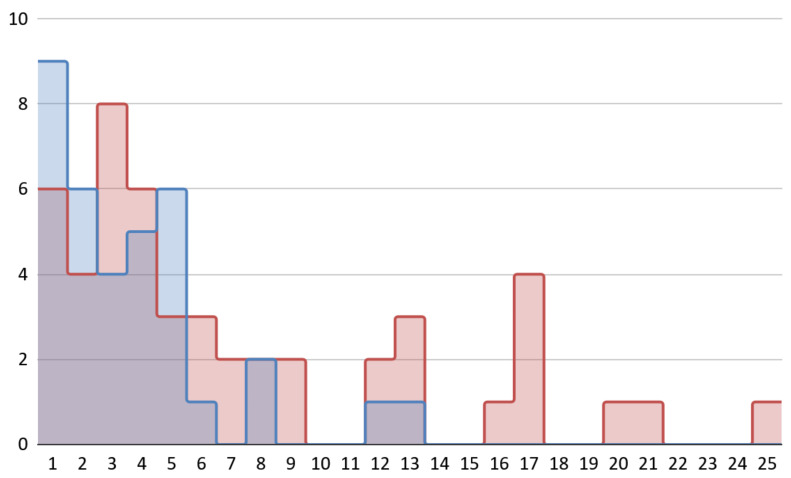
Distribution of HI scores at baseline (red) and at final follow-up (blue). The x-axis shows HI values, and the y-axis shows the number of patients in each bin.

The mean (SD) HI decreased descriptively from 7.0 (6.2) at tb to 2.5 (3.0) at tf, corresponding to a mean change of −4.4 (5.0) points (negative values indicate lower TMJ disease severity at follow-up). The paired Wilcoxon signed-rank test showed a statistically significant difference between HI values at tb and tf (*p* < 0.001). The distribution of HI scores at tb and tf is shown as overlaid histograms in Figure 1. This comparison is descriptive and should not be interpreted as evidence of treatment effectiveness in the absence of a comparator group.

### 3.3. Baseline-Adjusted Associations with Follow-Up HI

For OBC-21 items, the available sample size varied by item from n = 42 to n = 51 because occasional “unable to determine” responses were treated as missing for the specific item (available-case analysis per item). Overall missingness across OBC-21 responses was low (49/1071 observations, 4.6%).

Associations between baseline predictors and follow-up HI were evaluated using baseline-adjusted linear regression models with follow-up HI as the dependent variable and baseline HI included as an adjustment covariate. Separate models were fitted for each candidate predictor because the analysis retained an exploratory item-level structure and the sample size did not support construction of multivariable predictive models including multiple behavioral variables simultaneously.

Baseline HI was consistently associated with follow-up HI across models and showed a strong positive baseline-adjusted association with outcome (β = 0.32, 95% CI 0.22 to 0.41, *p* < 0.001; n = 51), indicating that baseline disease severity showed a strong association with follow-up HI and supporting its role as an adjustment variable in the analytic framework.

Symptom duration prior to treatment was not meaningfully associated with follow-up HI after adjustment for baseline HI (β = 0.02, 95% CI −0.10 to 0.15, *p* = 0.70; n = 48).

Similarly, injection laterality (bilateral vs. unilateral) was not meaningfully associated with follow-up HI in baseline-adjusted analyses (β = −0.42, 95% CI −1.73 to 0.89, *p* = 0.52; n = 51).

**Figure 2:**
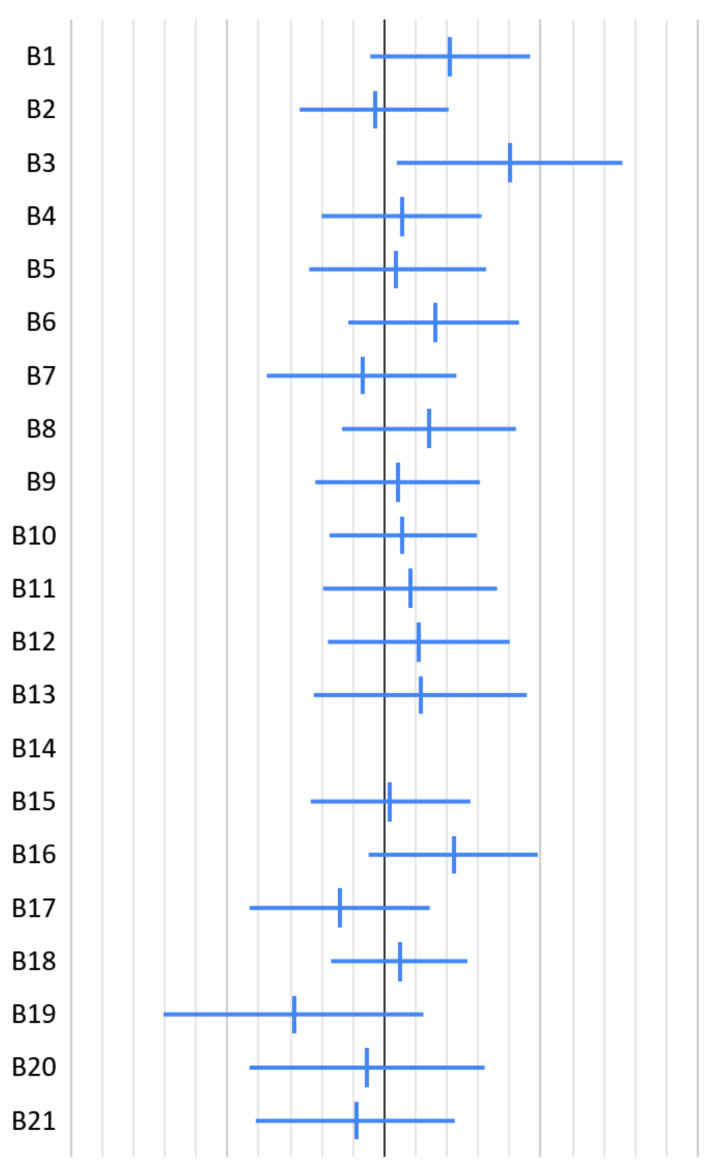
Baseline-adjusted regression coefficients for associations between baseline OBC-21 item scores and follow-up HI, with 95% confidence intervals. Points indicate unstandardized regression coefficients (β), horizontal lines show 95% confidence intervals, the black vertical line marks no association, and gridlines are spaced at 0.2. Behavioral labels: B1, sleep bruxism; B2, sleep posture; B3, awake bruxism; B4, clenching; B5, tooth contact; B6, muscle tension; B7, jaw shifting; B8, tongue pressing; B9, tongue interposition; B10, cheek biting; B11, jaw bracing; B12, object biting; B13, gum chewing; B14, instrument playing; B15, chin resting; B16, unilateral chewing; B17, snacking; B18, prolonged speaking; B19, singing; B20, yawning; B21, phone cradling. B14 was not estimable because this item showed no variability at baseline.

Among the 21 baseline OBC-21 items, no clear baseline-adjusted associations with follow-up HI were identified after correction for multiple exploratory comparisons (Table 1, Figure 2). The largest absolute regression coefficients were observed for awake bruxism (B3), singing (B19), unilateral chewing (B16), sleep bruxism (B1), and muscle tension without tooth contact (B6), but these estimates were small to modest in magnitude and imprecise. Awake bruxism (B3) showed a nominal association with follow-up HI before multiplicity correction (β = 0.80, 95% CI 0.08 to 1.52, *p* = 0.03), but this association did not remain significant after false discovery rate adjustment (FDR-adjusted *p* = 0.60). The remaining behavioral items showed no clear baseline-adjusted associations with follow-up HI. Instrument playing (B14) could not be evaluated because the item showed no variability at baseline.

## 4. Discussion

### 4.1. Principal Findings

This secondary exploratory cohort analysis examined whether baseline oral behaviors measured with the OBC-21 were associated with follow-up TMJ disease severity quantified by the HI in patients treated with intra-TMJ I-PRF, after adjustment for baseline HI values. The interpretation of the findings was based on baseline-adjusted regression models with follow-up HI as the outcome and baseline HI included as an adjustment covariate. The aim was not to re-estimate overall treatment effectiveness, but rather to explore whether selected baseline behavioral features might help explain heterogeneity in follow-up TMJ disease severity within this cohort. Overall, baseline oral behaviors showed no strong baseline-adjusted associations with follow-up HI.

In baseline-adjusted regression analyses using follow-up HI as the outcome, individual OBC-21 items showed no clear baseline-adjusted item-level associations after accounting for baseline disease severity, supporting the interpretation that behavioral variables did not emerge as strong standalone baseline-adjusted factors associated with follow-up HI in this treated TMJ cohort. Among the 21 OBC-21 items, the largest absolute baseline-adjusted regression coefficients were observed for awake bruxism, singing, unilateral chewing, sleep bruxism, and muscle tension without tooth contact, but these associations were small to modest in magnitude, imprecisely estimated, and did not remain significant after correction for multiple comparisons. Symptom duration and injection laterality were not meaningfully associated with follow-up HI after adjustment for baseline HI.

Directly comparable prognostic studies in injection-treated TMJ cohorts are scarce. In a prospective single-arm study of TMJ osteoarthritis treated with a single intra-articular crosslinked hyaluronic acid injection, Baron et al. reported that patient satisfaction at 6 months was associated mainly with pain-related measures and pain reduction, whereas bruxism and symptom duration were not retained as significant variables in multivariable analysis [43]. Although that study differed from the present one in intervention, diagnosis, and outcome definition, it is broadly consistent with the present finding that symptom duration did not meaningfully explain follow-up TMJ disease severity and that oral-behavior variables did not emerge as strong standalone baseline-adjusted factors associated with follow-up HI in this treated TMJ cohort.

### 4.2. Interpretation of the Exploratory Associations

In the exploratory item-level results, several baseline oral behaviors showed larger absolute regression coefficients than others, although none remained statistically significant after correction for multiple comparisons. Because the primary analyses used baseline-adjusted regression models with follow-up HI as the outcome, these estimates should be interpreted as preliminary signals rather than evidence of independently adjusted behavioral associations.

First, residual baseline severity dimensions not fully captured by total baseline HI may still have shaped apparent associations between baseline oral behaviors and follow-up status. For this reason, the primary analyses were based on follow-up HI adjusted for baseline HI rather than on unadjusted change-based association analyses. Under this framework, the observed directional patterns should be interpreted as exploratory signals potentially influenced by residual baseline severity dimensions. A related observation has been reported in other TMJ intervention settings; for example, Castaño-Joaqui et al. found that higher baseline pain was associated with worse long-term oral-health-related quality-of-life outcomes after TMJ arthrocentesis with hyaluronic acid [44].

Second, the OBC-21 reflects behavior frequency over the preceding week and may therefore capture short-term symptom-related states rather than stable behavioral traits. This interpretation is consistent with cross-sectional TMD literature showing that oral behaviors cluster with pain, jaw dysfunction, and psychosocial burden. Xu et al. reported significant associations between oral behaviors and jaw functional limitation, anxiety, and depression in patients with TMD [45]. More recently, Yap et al. found that comorbid depression and anxiety were linked to greater pain, sleep propensity, jaw dysfunction, and oral behaviors in TMD patients, reinforcing the view that oral behaviors may be embedded within broader symptom and psychosocial profiles rather than acting as isolated mechanical exposures [46]. In a related younger TMD population, jaw overuse behavior was also associated with poorer oral-health-related quality of life [47].

Third, several behaviors may function as proxies for broader patient phenotypes or contextual factors not measured in the present dataset. This interpretation is also compatible with validation data showing that OBC scores are strongly related to pain intensity, TMD severity indices, anxiety/depression, and oral-health-related quality of life [48].

Fourth, in baseline-adjusted analyses, awake bruxism showed one of the largest positive regression coefficients, whereas singing showed one of the largest negative coefficients among the examined behaviors. Although these estimates did not remain statistically significant after correction for multiple comparisons, they should not be overinterpreted. In particular, the negative coefficient for singing should not be interpreted as evidence of a favorable or protective behavioral pattern. These item-level estimates may reflect residual confounding, selection effects, baseline symptom structure, or chance variation in a modest sample.

Taken together, the observed associations should not be interpreted causally. Rather, they should be regarded as hypothesis-generating observations that require more rigorous evaluation in larger, better-characterized cohorts, with explicit modeling of baseline disease severity and stratification by diagnostic subgroup or structural disease stage.

### 4.3. Symptom Duration and Injection Laterality

Symptom duration was not meaningfully associated with follow-up HI after adjustment for baseline HI in this cohort. This suggests that, within the observed range and under the present measurement approach, chronicity alone did not explain variation in outcome. This is in line with the study by Baron et al., in which symptom duration was not associated with treatment satisfaction after intra-articular hyaluronic acid injection [43]. However, symptom duration is a crude summary measure that does not distinguish between intermittent and persistent symptoms, prior exacerbations, previous treatments, or changes in diagnosis over time. More detailed characterization of disease course may therefore prove more informative than duration alone in future prognostic analyses.

Injection laterality, classified as unilateral versus bilateral, was also not associated with follow-up HI after adjustment for baseline HI. This may indicate that laterality pattern did not materially influence follow-up TMJ disease severity once baseline disease severity had been taken into account. Alternatively, the absence of association may reflect the use of a composite outcome that does not isolate side-specific changes, particularly in patients with bilateral symptoms, or limited precision in a modest sample. Comparator literature is limited in this area, because published prognostic studies in TMJ injection cohorts have mainly emphasized pain, satisfaction, function, or quality-of-life outcomes rather than side-specific response patterns [43,49].

### 4.4. Outcome Considerations

The HI provided a pragmatic and clinically interpretable measure of TMJ disease severity at baseline and follow-up and allowed evaluation of baseline-adjusted associations with follow-up status. In this study, change from baseline was presented only as a descriptive summary of clinical evolution, whereas the primary association analyses were based on follow-up HI adjusted for baseline HI. This distinction is important because baseline-adjusted models are less susceptible than unadjusted change-based analyses to distortion related to baseline severity structure, mathematical coupling, and regression to the mean.

The broader TMJ literature also suggests that prognostic conclusions may vary depending on the chosen outcome domain. Baron et al. focused on satisfaction, pain while chewing, and mandibular opening after HA injection [43], whereas Su et al. developed prognostic models for follow-up oral-health-related quality of life after arthrocentesis with HA in TMJ osteoarthritis [49]. In a retrospective cohort study, Castaño-Joaqui et al. reported long-term improvement in oral-health-related quality of life after TMJ arthrocentesis with hyaluronic acid and found worse follow-up quality-of-life outcomes in patients with higher baseline pain [44]. In a PRP-treated cohort, Sikora et al. showed that patient-reported TMD burden correlated extensively with other clinical and subjective variables, underscoring the multidimensional nature of treatment response [50].

In addition, the HI is derived from component scores with limited response categories, which may reduce sensitivity to detecting small but clinically relevant changes and increase the frequency of tied values. This may be particularly relevant when follow-up scores cluster toward the lower end of the scale, as observed here. Because HI is a bounded, integer-valued score and follow-up values clustered toward the lower end of the scale, linear regression was used only as an exploratory approximation. Residual diagnostics did not indicate major deviations that would preclude this approach, but the findings should be interpreted cautiously and not as confirmatory model-based evidence. Future studies may therefore benefit from complementing the HI with more granular measures of pain, function, and patient-reported symptoms.

### 4.5. Missing Data and Item-Level Estimation

Item-level analyses of OBC-21 predictors were based on available cases because responses marked as “unable to determine” were treated as missing for the respective item. Overall missingness across OBC-21 responses was low (4.6%), suggesting that available-case estimation was unlikely to materially bias item-level association estimates, although it contributed to predictor-specific differences in precision. As a result, sample size varied across items and may have limited the ability to detect modest associations. In addition, one item (instrument playing) could not be evaluated because it showed no variability at baseline. These findings highlight a practical limitation of item-level exploratory analyses in modest samples: some candidate predictors may be uncommon, unevenly distributed, or measured with limited informational value for regression-based association analyses in this clinical population.

The current analysis provides an early exploratory signal from a distinct and clinically relevant treatment setting rather than a direct replication of previous association studies, and its value lies primarily in identifying candidate behavioral domains for further study in similarly treated cohorts.

### 4.6. Multiple Comparisons and Cautious Inference

The item-level analysis included 21 OBC-21 predictors, creating substantial multiplicity. In this context, some nominally significant or borderline-significant findings before multiplicity correction would be expected by chance alone. Several relatively large regression coefficients were observed but did not remain statistically significant after correction for multiple comparisons and should therefore be interpreted as exploratory signals rather than robust baseline-adjusted associations with follow-up HI.

This cautious framing is especially important because stronger-looking associations between oral behaviors and TMD outcomes have generally come from cross-sectional studies or from formal prognostic modeling rather than from small exploratory baseline-adjusted single-predictor analyses such as those used in the present study. Xu et al. and Yap et al. reported cross-sectional associations between oral behaviors and psychosocial or functional burden, while Su et al. developed explicit prediction models in which awake bruxism, sleep bruxism, and chewing-side preference contributed to the prediction of follow-up oral-health-related quality of life [45,46,47,49]. By contrast, the present study was not designed as a multivariable prediction-modeling study and should not be interpreted at the level of multivariable prognostic modeling.

### 4.7. Clinical Implications

The present findings do not support strong claims about specific oral behaviors as standalone baseline-adjusted factors associated with follow-up HI after intra-TMJ I-PRF therapy. Nevertheless, they suggest that some behavioral domains may merit prioritization for confirmatory evaluation in future studies. In a retrospective cross-sectional study, high oral behavior levels and OBC scores were associated with chronic painful TMD, and sleep bruxism emerged as the strongest predictor of TMD pain [51].

From a pragmatic clinical standpoint, such behaviors may still be relevant as part of conservative TMD management strategies, but the present analysis does not establish that they independently determine follow-up TMJ disease severity in this setting. A restrained interpretation is therefore warranted: oral behaviors may be useful as candidate markers for phenotyping and counseling but not yet as validated standalone markers of follow-up status.

### 4.8. Strengths and Limitations

This study has several strengths. The cohort was consecutive, all included participants had baseline and final follow-up HI data available, follow-up for the analyzed outcome was complete, resulting in complete outcome data for the primary analysis, and oral behaviors were recorded systematically using a standardized checklist. The use of baseline-adjusted regression models was appropriate for evaluating associations between ordinal predictor scores and follow-up HI. Use of the OBC is also supported by external literature showing meaningful relationships with pain, TMD severity, psychosocial variables, and quality-of-life measures in TMD populations [45,48].

At the same time, several limitations should be acknowledged. This was a secondary exploratory observational analysis conducted within a trial-derived cohort. The sample size was modest for exploratory baseline-adjusted association analyses, particularly given the number of item-level analyses performed. Associations were examined primarily in separate baseline-adjusted regression models rather than in multivariable models incorporating multiple behavioral variables simultaneously within a single multivariable analytic framework. In addition, the analyses were not adjusted for additional clinical, structural, or psychosocial covariates that may influence follow-up HI. In addition, the absence of a comparator group limited the ability to evaluate treatment-specific effect modification. Although change from baseline was summarized descriptively, the primary analytic framework was based on baseline-adjusted follow-up models. Oral behaviors were self-reported over a short recall window and may reflect transient symptom states as much as stable traits. Finally, item-level missingness reduced precision and produced unequal analytic sample sizes across predictors.

### 4.9. Generalizability

The generalizability of these findings is limited. The study was conducted in a single clinical center and included consecutive adult patients selected for intra-TMJ I-PRF treatment according to local clinical indications within routine clinical practice. Accordingly, the results may not generalize to pediatric populations, patients managed non-invasively, other injectable therapies, or cohorts with different diagnostic composition, healthcare settings, or severity distributions. Baseline-adjusted associations observed in this cohort may therefore not be directly transportable to populations with different treatment pathways or baseline severity profiles. This is particularly relevant because the comparator literature spans heterogeneous populations, including TMJ osteoarthritis treated with hyaluronic acid, internal derangement treated with arthrocentesis plus hyaluronic acid, broader TMD cohorts assessed cross-sectionally, and PRP-treated open-label cohorts, all of which differ from the present case-mix, treatment framework, and outcome definition [43,44,45,46,47,49,50,51].

### 4.10. Future Directions

Future studies should prespecify a limited set of candidate oral behaviors for confirmatory testing, preferably in larger cohorts with sufficient power and explicit handling of multiplicity. Analyses should account for baseline HI directly, for example by modeling follow-up HI adjusted for baseline severity, as implemented in the present exploratory analyses. More detailed phenotyping—including diagnosis strata, imaging findings, pain intensity, functional limitation, and psychosocial measures—would help clarify whether oral behaviors act as mechanistic contributors, behavioral consequences, or markers of patient subgroups. Repeated assessment of oral behaviors over time may also help determine whether changes in such behaviors track with changes in TMJ disease severity over follow-up.

## 5. Conclusions

In this secondary exploratory cohort analysis, baseline disease severity showed the strongest association with follow-up HI, whereas symptom duration, injection laterality, and individual OBC-21 items did not show robust baseline-adjusted associations with follow-up HI after intra-TMJ I-PRF therapy and correction for multiple comparisons. The nominal and directional item-level patterns, including those for awake bruxism and singing, should be interpreted only as hypothesis-generating and may reflect residual baseline severity dimensions not fully captured by total baseline HI, baseline symptom structure, selection effects, or chance variation in a modest sample. Larger, better-characterized cohorts are needed to confirm whether selected behavioral domains have prognostic relevance in this treatment setting.

## Figures and Tables

**Table 1:** Baseline-adjusted associations between OBC-21 item scores at baseline and follow-up Helkimo Index (HI).

Candidate Predictor	n	β	Lower 95% CI	Upper 95% CI	*p* Value	FDR-Adjusted *p*
B1: Sleep bruxism	42	0.42	−0.09	0.93	0.107	0.743
B2: Sleep posture	49	−0.06	−0.54	0.41	0.787	0.829
B3: Awake bruxism	50	0.80	0.08	1.52	0.030	0.597
B4: Clenching	49	0.11	−0.40	0.62	0.661	0.829
B5: Tooth contact	47	0.08	−0.48	0.65	0.772	0.829
B6: Muscle tension	46	0.32	−0.23	0.86	0.249	0.829
B7: Jaw shifting	48	−0.14	−0.75	0.46	0.637	0.829
B8: Tongue pressing	47	0.28	−0.27	0.84	0.307	0.829
B9: Tongue interposition	48	0.09	−0.44	0.61	0.745	0.829
B10: Cheek biting	50	0.12	−0.35	0.59	0.614	0.829
B11: Jaw bracing	44	0.17	−0.39	0.72	0.552	0.829
B12: Object biting	51	0.22	−0.36	0.80	0.447	0.829
B13: Gum chewing	51	0.23	−0.45	0.91	0.496	0.829
B14: Instrument playing	51	Not estimable	Not estimable	Not estimable	—	—
B15: Chin resting	50	0.04	−0.47	0.55	0.882	0.882
B16: Unilateral chewing	49	0.44	−0.10	0.98	0.111	0.743
B17: Snacking	49	−0.28	−0.86	0.29	0.326	0.829
B18: Prolonged speaking	51	0.10	−0.34	0.53	0.654	0.829
B19: Singing	50	−0.58	−1.41	0.25	0.165	0.826
B20: Yawning	50	−0.11	−0.86	0.64	0.762	0.829
B21: Phone cradling	50	−0.18	−0.82	0.45	0.565	0.829

Baseline-adjusted linear regression models with follow-up HI as the dependent variable and baseline HI as an adjustment covariate were used. FDR-adjusted *p* values are reported to account for multiple exploratory item-level comparisons. β—unstandardized regression coefficient; n—sample size; CI—confidence interval; FDR—false discovery rate.

## Data Availability

The data presented in this study are available from the corresponding author upon reasonable request. The trial protocol is available at ClinicalTrials.gov (Identifier: NCT05883982, registered on 22 May 2023).

## Abbreviations

The following abbreviations are used in this manuscript:

HI	Helkimo Index
I-PRF	Platelet-Rich Fibrin
OBC-21	Oral Behavior Checklist-21
PRP	Platelet-Rich Plasma
TMDs	Temporomandibular Joint Disorders
TMJ	Temporomandibular Joint
